# Knowledge and practices of general practitioners regarding psychiatric problems

**DOI:** 10.4103/0972-6748.57853

**Published:** 2009

**Authors:** R. K. Chaudhary, B. P. Mishra

**Affiliations:** Department of Psychiatry, Dayanand Medical College and Hospital, Ludhiana - 141001, Punjab, India; 1 Department of Clinical Psychology, Dayanand Medical College and Hospital, Ludhiana - 141001, Punjab, India

**Keywords:** Attitude, Knowledge, Treatment practices

## Abstract

**Background:**

Mental health problems account for 12% of global disease burden and non-psychiatrist medical practitioners deal with a large proportion of this burden. This study was planned to assess the knowledge, attitude and treatment practices of non-psychiatrist medical practitioners regarding mental health problems.

**Materials and Methods:**

One hundred Allopathic and 25 each of Homeopathic and Ayurvedic medical practitioners were interviewed and assessed using a semi-structured performa.

**Results:**

Majority (95%) of them were aware regarding etiology, increasing incidence and treatment facilities available for mental health problems. Treatment modalities include counseling and medication but 69.9% of them had not received any formal training in administering them.

**Conclusions:**

98.5% practitioners providing mental health services at the primary level feel the need to be properly trained and oriented in the management of these patients to improve quality of healthcare.

As per the World Health Report of 1995, about 500 million people are believed to suffer from neurotic, stress-related and somatoform disorders. World Health Report 2001, which is dedicated to the theme of mental health, shows that disorders are estimated to account for about 12% of the global burden of disease and also represent four of the ten leading causes of disability worldwide. As WHO has shifted its emphasis from prevalence rates to the concept of DALY -“Disability Adjusted Life Years”, the Neuropsychiatric Disorders rank very high on the list of global burden of disease.

India with a population of more than a billion, houses one of the highest number of mentally ill persons who require long-term care. With less than 10% availability of the inpatients’ care required for patients with mental health problems and less than one psychiatrist available for one lakh Indians, the gap between resources and requirements remains too broad (Trivedi, 2002).

Due to this wide gap, a large number of psychiatric patients do not receive adequate treatment and suffer from longstanding illness and resulting disability. A large portion of the patients who do ultimately reach the psychiatry outdoor, reach late, when the illness has already become chronic and resistant to therapy. The issue of less number of psychiatrists is further compounded by the striking ignorance about and lack of adequate skills for treating patients with mental health problems among the general care physicians and members of other medical sub-specialties.

There is a wide gap between the mental health needs of the community and the available psychiatric services in India (Neki, 1973). The psychiatric morbidity among the clients of general practitioners has been reported to be ranging between 10 to 36% (Murthy *et al*., 1981) and 27% among clients of general hospital outpatients (Murthy and Wig, 1977). In a study of 200 GPs in Bangalore, (Shamasunder, 1978) reported that 65% general practitioners (GPs) found psychiatric morbidity less than 10% in their practice while 24% reported a figure of less than 20%. This reflects the degree to which the GPs are aware about mental illness.

Not much work has been done in India regarding the opinions and attitudes of non-psychiatrist medical practitioners towards mental illness. Keeping this in view, the present study was planned.

## MATERIALS AND METHODS

The study was carried out in the city of Ludhiana and its surrounding areas/satellite towns in a radius of 20 km. The sample consisted of 158 non-psychiatrist medical practitioners (100 Allopathic, 33 Homeopathic and 25 Ayurvedic). Only qualified registered medical practitioners were included in the study. The data was collected over a period of 12 months.

All the practitioners were contacted and visited personally. The aims and objective of carrying out the survey was explained to them through a written letter as well as personally, on meeting.

A detailed questionnaire prepared for the survey was administered and got filled by the medical practitioner, at his/her convenience and in case it was required, the questionnaire was left with the medical practitioner, to be returned back by post in a pre-addressed and stamped envelope. Strict confidentiality was employed in carrying out the survey and in the use of information provided by each respondent.

The information collected was analyzed in the domains of the knowledge, attitude and treatment practices of non-psychiatrist medical practitioners with regard to mental health (psychiatric) problems. The observations from this study were used to comment on the availability of the existing mental health services and to find the adequacy or the deficiencies in these services.

## DISCUSSION

A total of 158 non-psychiatrist practitioners were surveyed, out of which 133 practitioners answered the questionnaire, forming a response rate of 84.17% which is much more as compared with 41% and 62% in works of similar kind (Fauman, 1981 & 1983) and also more than that reported by Chad and Shome (1996) in which the response rate was 63.9% [Table 1].

**Table 1:** Sociodemographic characteristics of general practitioners (N = 133)

	(%)
Age (in years)	
<30	17 (12.78)
31–40	39 (29.32)
41–50	43 (32.33)
51–60	34 (25.56)
Sex	
Male	101 (75.94)
Female	32 (24.06)
System of medicine in which practicing	
Homeopathy	31 (23.31)
Ayurvedic	19 (14.29)
Allopathy	83 (62.41)
Qualification	
Graduate	104 (78.20)
Postgraduate	29 (21.80)
Years spent in private practice	
Less than 5	11 (8.27)
5–10	36 (27.07)
10–20	43 (32.33)
More than 20	43 (32.33)
Type of practice	
General practice (GP)	109 (81.95)
Specialist practice	24 (18.05)
Service provided	
Outpatient consultation	93 (69.92)
Both (Outpatient and inpatient)	40 (30.07)
Any training received in dealing with patients suffering from mental health (Psychiatric) problems	
Yes (N1)	97 (72.93)
No (N2)	36 (27.07)
When was the training received (N1)	
Internship	67 (69.08)
Graduation	30 (30.92)
Percentage of practitioners who continued to update their knowledge in dealing with mental health problems	73 (75.26)

Around 88.7% of the practitioners admitted seeing patients with mental health problems in their practice which is in consistent with a survey done on general practitioners in Jaipur city (Gautam S, Gupta I.D, Kamalpreet, 1992).

While tapping the knowledge and attitudes [Table 2] of the general practitioners, 70.68% felt mental health problems are very common. 89.47% reported that these are due to a combination of stress, social, cultural, individual, biological and organic factors, which is in accordance with findings of Gautam S (1992) where the response rate was 90%. Almost all the practitioners reported that mental illness is of serious concern. This confirms the findings of Verghese and Beig (1974) who reported that the majority of the people have a positive attitude towards mental illnesses.

**Table 2:** Knowledge and attitude regarding mental health (psychiatric) problems (N =133)

	(%)	
Do you feel mental health (psychiatric) problems are		
Very common	94 (70.68)	
Common	39 (29.32)	
What do you think are the cause(s) for mental health problem		
Stress of different kinds	14 (10.53)	
Combination of (stress, social, cultural, individual, biological and organic factors)	119 (89.47)	
Do you think	Yes	No
People of all ages can have mental health problems	132 (99.3)	1 (0.7)
Children can have mental health problems	131 (98.5)	2 (1.5)
Old age people can have mental health problems	132 (99.3)	1 (0.7)
Mental health problems are illnesses at all	132 (99.3)	1 (0.7)
Mental health problems are worthy of serious concern and attention	133 (100)	-
Mental health problems are treatable	133 (100)	-
These problems can improve if nothing is done to treat them is done to treat them	11 (8.3)	122 (91.7)
Do you think patients with mental health problems deserve to have equal human rights (including that of marriage)		
Yes	110 (82.71)	
No	4 (3)	
Not sure	19 (14.29)	
What is your response and reaction when you come across a person with a mental health problem		
Sympathy and help	133 (100)	

The majority of patients (82.7%) seen by general medical practitioners were having psychosomatic problems like sleep problems (84.2%); appetite disturbances (84.2%); and abnormal and irrational fear (84.2%); followed by mood disturbances and problems in sexual activity (72.9%) [Table 3]. A considerable number of drug addicts (62.41%) and problems of forgetfulness (66.17%) were also seen by general practitioners.

**Table 3:** Practitioners seeing patients with the following symptomatology (N =133)

Problem	Not seeing (%)	Frequently seeing (%)	Occasionally seeing (%)
Disturbances in mood	3 (2.25)	110 (82.7)	20 (15.04)
Attempted suicide/suicidal intent	34 (25.56)	13 (9.77)	86 (64.66)
Sleep problems	4 (3)	112 (84.2)	17 (12.78)
Appetite disturbance	2 (1.5)	112 (84.2)	19 (14.3)
Disturbance in sexual activity	10 (7.52)	97 (72.9)	26 (19.55)
Excessive ghabrahat/anxiety/worries	11 (8.2)	84 (63.16)	38 (28.57)
Abnormal and irrational fears	4 (3)	112 (84.2)	17 (12.78)
Physical somatic complaints not due to physical illness	7 (5.26)	82 (61.65)	44 (33.08)
Fits/attacks of unconsciousness due to epilepsy	4 (3)	97 (72.93)	32 (24.06)
Fits/attacks of unconsciousness not due to epilepsy	18 (13.53)	71 (53.38)	44 (33.08)
Memory impairment and forgetfulness	10 (7.52)	88 (66.17)	35 (26.32)
Problems related to drugs/bhang/smack/alcohol/opium	12 (9)	83 (62.41)	38 (28.57)
Marital discord and adjustment problems	11 (8.27)	59 (44.36)	63 (47.37)
Children/adolescents with			
Developmental delays/speech problems	9 (6.77)	49 (36.84)	75 (56.39)
Behaviour problems	6(4.5)	57 (42.9)	70 (52.6)

Majority of non-psychiatrist medical practitioners (79.7%) do not know any diagnostic criteria neither have they any exposure or training to deal with mental illness [Table 4]. They treat their psychiatric patients on their own intuitions. This finding is in accordance with Gautam and Kapur (1980) where they reported 71.7% of general medical practitioners without knowledge and training.

**Table 4:** Treatment practices regarding mental health (psychiatric) problems (N =133)

	(%)
Do you know any diagnostic criteria used for diagnosis	
Yes	27 (20.30)
No	106 (79.70)
Whether there is rile of blood tests, EEG, CT Scan/MRI in establishing/confirming diagnosis	
Yes	99 (74.4)
No	34 (25.6)
Do you like to treat the mental health problems in your patients	
Yes	124 (93.23)
No	9 (6.77)
How long do you like to treat the patient yourself before seeking the help of a psychiatrist	
1 week	11 (8.27)
1 month	9 (6.77)
1–3 months	3 (2.25)
3–6 months	3 (2.25)
Varies from patient to patient	107 (80.5)
How do you treat your patients	
By medication	7 (5.26)
Combination of counseling and medication	118 (88.72)
No answer	8 (6)
Any training received in above modalities	
Yes	40 (30.1)
No	93 (69.9)
Medications commonly prescribed	
Anxiolytics	5 (3.76)
Homeopathy medicines	3 (2.25)
Combination of (antidepressants, anxiolytics, antipsychotics, sedatives, anticonvulsants)	125 (93.9)

As far as psychiatric referrals are concerned, 38.3% practitioners reported that they refer the patient to a psychiatrist when required, but 34.6% reported that they refer only if it is unavoidable and symptoms are not controlled, whereas 27% refer occasionally, at will. Majority of the practitioners sought psychiatric consultation when needed which was in accordance with the findings reported by Narang, R.L. and Gupta, Rajeev (1987) as 75%, and Chadda and Shome (1996) reported 66%. Approximately half of the practitioners (50.3%) reported that patients accept their advice about psychiatric referral with reluctance, showing social stigma. This finding is in accordance with the findings of Chadda and Shome (1996) where 45% patients were reluctant regarding referrals. Some of the practitioners (4.5%) reported that patients used to refuse to go to a psychiatrist and 2.25% of the practitioners reported dropout of the treatment when referred. This finding also supports the finding reported by Chadda and Shome (1996), where the patients refused to comply with advice about psychiatric referral according to 8% of practitioners [Table 5].

**Table 5:** Experience of non-psychiatrist medical practitioners with psychiatry referral (N =133)

	(%)
Whether referring the patients to a psychiatrist when required	
Always	51 (38.3)
Sometimes	36 (27.1)
Only if unavoidable	46 (34.6)
Whether informing the patient and attendants about psychiatric referral	
Yes	133 (100)
Reaction of the patient on being referred to a psychiatrist	
Easy acceptance	57 (42.8)
Acceptance with reluctance	67 (50.3)
Refusal to accept	6(4.5)
Not known, as patient drops treatment	3 (2.25)
If refuses to accept, do you try to educate regarding the need of referral	
Always	117 (87.97)
Sometimes	16 (12.03)
Percentage of patients consulting the psychiatrist and coming back to you	
<25%	67 (50.4)
25–50%	40 (30.1)
>50%	26 (19.5)
Whether getting the feedback from psychiatrist of referred patients	
Yes	66 (49.6)
No	67 (50.4)
Mode of communication	
Telephonic communication	27 (40.9)
A written report	20 (30.3)
Patient’s own feedback	9 (13.63)
By all above means	10 (15.2)
Opinion about usefulness of psychiatric referral	
Always helpful	96 (72.2)
Helpful sometimes	35 (26.3)
Not sure	2 (1.5)

Regarding the feedback from the psychiatrist of the referred patient, less than half (49.6%) of the practitioners received the feedback. Somewhat similar results have been described in earlier literature (Pullen, 1993) and also in a survey done on practitioners by Chadda and Shome (1996). Regarding the usefulness of the psychiatric referral, majority (72.2%) of the practitioners reported that the referral is always helpful. Somewhat similar findings were seen in a survey by Chadda and Shome (1996), when the response rate was 90%.

Almost all the non-psychiatrist medical practitioners in this study agreed that the incidence of mental health problems is increasing in the general population which is also in accordance with the findings reported by Narang, R.L. and Gupta Rajeev (1987).

Regarding the availability of psychiatric services, majority of the practitioners (66.9%) reported that they are not sufficient. Many epidemiological studies (Sethi *et al*., 1967) and Neki’s (1973) findings support this notion. Majority (98.5%) of the practitioners felt that they themselves and other practitioners need to know more about the psychiatric problems and the treatment available as the training received by them during their training period was only for two to three weeks. These findings are in accordance with the findings of a survey done on family physicians where 46% of the respondents felt that they were dissatisfied with their competence to treat psychiatric illness (Fisher *et al*., 1973). The finding is also in accordance with Narang R.L. and Gupta Rajeev (1987) where about 70% practitioners reported the same [Table 6].

**Table 6:** Opinion regarding mental health (psychiatric) problems and services in city of Ludhiana and its surrounding areas (N =133)

Do you think	Yes (%)	No (%)	Not sure (%)
Incidence of mental health problems is increasing in general population	133 (100)	-	-
Available services are sufficient	41 (30.8)	89 (66.9)	3(2.25%)
There is adequate number of trained psychiatrists	52 (39.1)	79 (59.4)	2 (1.5)
Trained child psychiatrists	15 (11.3)	114 (85.7)	4(3%)
Trained old age psychiatrists	20 (15.03)	108 (81.2)	5 (3.7)
Trained clinical psychologists	21 (15.8)	107 (80.5)	5 (3.7)
Trained counselors/therapists	15 (11.3)	113 (84.9)	5 (3.7)
Specialized centres/hospitals	32 (24.1)	99 (74.7)	2 (1.5%)
The non-psychiatrist medical practitioners are utilizing the expertise services properly	32 (24.1)	95 (71.4)	6(4.5%)
The medical practitioners need to know more about the psychiatric problems and treatment available	131 (98.5)	2 (1.5)	-
You need to know more about the psychiatric problems and their management	130 (97.7)	3 (2.25)	-
The mental health services need to improve further	131 (98.5)	2 (1.5)	-

## CONCLUSION

In this study, it was found that the majority of the non-psychiatrist medical practitioners see patients with mental health (psychiatric) problems in their practice. Majority of them (79.7%) do not know any diagnostic criteria used for diagnosis of mental health problems. They are aware of the etiology, increasing incidence and treatment facilities available for mental health problems. They treat the patients with medication and counseling but the majority of them have not received any formal training in these fields. Majority of the practitioners felt that the existing mental health services are not sufficient to meet the needs of the people. So, we concluded that there is a lack of training of general practitioners in dealing with patients having mental health (psychiatric) problems and there is need for further improvement of the existing mental health services.
